# A Study of Urban Haze and Its Association with Cold Surge and Sea Breeze for Greater Bangkok

**DOI:** 10.3390/ijerph20043482

**Published:** 2023-02-16

**Authors:** Nishit Aman, Kasemsan Manomaiphiboon, Natchanok Pala-En, Bikash Devkota, Muanfun Inerb, Eakkachai Kokkaew

**Affiliations:** 1 The Joint Graduate School of Energy and Environment, King Mongkut’s University of Technology Thonburi, Bangkok 10140, Thailand; 2 Center of Excellence on Energy Technology and Environment, Ministry of Higher Education, Science, Research and Innovation, Bangkok 10140, Thailand; 3 Pollution Control Department, Ministry of Natural Resources and Environment, Bangkok 10400, Thailand; 4 Faculty of Technology and Environment, Prince of Songkla University (Phuket Campus), Phuket 83120, Thailand

**Keywords:** urban haze, haze type, sea breeze, cold surge, Greater Bangkok

## Abstract

This study deals with haze characteristics under the influence of the cold surge and sea breeze for Greater Bangkok (GBK) in 2017–2022, including haze intensity and duration, meteorological classification for haze, and the potential effects of secondary aerosols and biomass burning. A total of 38 haze episodes and 159 haze days were identified. The episode duration varies from a single day to up to 14 days, suggesting different pathways of its formation and evolution. Short-duration episodes of 1–2 days are the most frequent with 18 episodes, and the frequency of haze episodes decreases as the haze duration increases. The increase in complexity in the formation of relatively longer episodes is suggested by a relatively higher coefficient of variation for PM_2.5_. Four meteorology-based types of haze episodes were classified. Type I is caused by the arrival of the cold surge in GBK, which leads to the development of stagnant conditions favorable for haze formation. Type II is induced by sea breeze, which leads to the accumulation of air pollutants due to its local recirculation and development of the thermal internal boundary layer. Type III consists of the haze episodes caused by the synergetic effect of the cold surge and sea breeze while Type IV consists of short haze episodes that are not affected by either the cold surge or sea breeze. Type II is the most frequent (15 episodes), while Type III is the most persistent and most polluted haze type. The spread of haze or region of relatively higher aerosol optical depth outside GBK in Type III is potentially due to advection and dispersion, while that in Type IV is due to short 1-day episodes potentially affected by biomass burning. Due to cold surge, the coolest and driest weather condition is found under Type I, while Type II has the most humid condition and highest recirculation factor due to the highest average sea breeze duration and penetration. The precursor ratio method suggests the potential effect of secondary aerosols on 34% of the total haze episodes. Additionally, biomass burning is found to potentially affect half of the total episodes as suggested by the examination of back trajectories and fire hotspots. Based on these results, some policy implications and future work are also suggested.

## 1. Introduction

Particulate matter (PM), having aerodynamic diameter ≤ 2.5 μm (PM_2.5_), is either emitted directly into the atmosphere from combustion, volcanic emission, sea spray, etc. (known as primary PM) or through gas-to-particle conversion by nucleation and complex multiphase chemical reactions in the atmosphere (known as secondary PM). In addition, primary and secondary PM can also undergo physical and chemical transformation and cloud processing [1,2]. In 2019, around 90% of the world’s population is exposed to an annual average PM_2.5_ level exceeding World Health Organization (WHO) Interim Target 1 (PM_2.5_ > 35 µg m^−3^) [3]. PM_2.5_ is a major health hazard that damages the respiratory and cardiovascular systems and contributes to premature mortality rates [4]. In addition, it also has direct and indirect impacts on weather and climate, and atmospheric visibility [1]. A prolonged existence of haze days (few days to weeks) in an urban environment (which is referred to as a haze episode) can be induced by the synergistic effect of multiple interrelated factors, which include anthropogenic emission, secondary aerosols [5,6], long–range transport [7,8,9], unfavorable local and synoptic meteorological conditions [10,11,12]. The effects of different meteorological processes on haze pollution are inconsistent and can have contrasting effects [11].

Bangkok, the capital city of Thailand, and its five surrounding provinces (Samut Prakan, Samut Sakhon, Nonthaburi, Nakhon Pathom, and Pathum Thani), which are collectively known as Greater Bangkok (GBK) is a major economic hub and an urban agglomeration in Southeast Asia. Haze pollution is frequently experienced in GBK during the dry season when the daily PM_2.5_ exceeds the daily National Ambient Air Quality Standard (NAAQS) of 50 µg m^−3^ several times per year as reported by the Pollution Control Department (PCD) [13]. This haze pollution is related to the emission from on-road vehicles [14,15,16,17], biomass burning [16,17,18,19,20], and unfavorable meteorological conditions induced by cold surges in Greater Bangkok [7,20].

Being a coastal metropolitan in Southeast Asia, GBK is under the influence of land–sea breeze, which can affect the transport and dispersion of air pollutants leading to improvement or worsening of the air quality as reported in other studies across the world [21,22,23,24]. Sea breeze can be modulated by topography [25], atmospheric stability [26], local prevailing winds, the Coriolis force [27], and interaction with synoptic winds [28,29,30]. Many studies have been carried out globally using observation data and modeling on sea breeze characterization [31,32,33] and its effects on air pollution with/without incorporating the effect of synoptic winds [22,34,35]. In Thailand, Phan and Manomaiphiboon (2012) [33] reported that sea breeze occurs very frequently over Rayong Province in Eastern Thailand during winter and can penetrate up to 25–55 km between early and mid-afternoon. A cold surge is another atmospheric phenomenon that affects weather and air pollution in major cities in Southeast Asia [7,36,37,38]. Ashfold et al. (2017) [36] reported elevated ozone and carbon monoxide during cold surges in Malaysia. Hien et al. (2011) [37] and Ly et al. (2018) [38] found that the arrival of cold surges in Hanoi city in Vietnam leads to an increase in the level of air pollutants after a few days. Similarly, Aman et al. (2020) [7] found that the selected haze episodes in GBK are initiated by the arrival of cold surges which leads to a decrease in temperature and relative humidity and the development of the temperature inversion layer. Wangwongchai et al. (2005) [39] reported heavy rainfall in Hat Yai due to cold surges in South Thailand. Wongsaming and Exell (2011) [40] developed the criteria for forecasting cold surges over Thailand. One important aspect of research in this direction is to understand the effect of the sea breeze and cold surge and their interaction on urban haze episodes in GBK which has not been well documented yet. Accordingly, this study aims to fill this knowledge gap by identifying haze episodes in GBK during 2017–2022, classifying them based on the cold surge and sea breeze, and investigating various characteristics of these haze episodes and types. Additionally, the potential effects of secondary aerosols and biomass burning have also been inspected.

## 2. Data and Methods

### 2.1. Study Area

This study focuses on GBK, situated in the lower part of Central Thailand with a registered population of 11 million and an area of 7762 km^2^ [41]. GBK is the economic center of Thailand which accounts for around 46% of the total gross domestic product (GDP) of Thailand [42]. The urban landscape is a complex mixture of commercial, residential, agricultural, and industrial areas [43], and the overall terrain is flat with an average height of less than 10 m (above mean sea level). It has a tropical wet climate, which is mainly governed by the northeast monsoon (November–February as the winter) and the southwest monsoon (May–October as the wet season) [44]. During the northeast monsoon, cool and dry air reaches Bangkok from continental mid-latitudes over which persistent strong high-pressure systems are present. The southeast monsoon brings moist air from the Gulf of Thailand and the Indian Ocean, which causes rain over a large part of Thailand. However, rain can be modulated at a sub-seasonal scale by intertropical convergence zone (ITCZ) movement along the north–south direction and tropical cyclones developed in the Western Pacific Ocean and North Indian Ocean. The transitional period of March–April between two monsoons is relatively warmer than other months and corresponds to the summer season. During winter, an important synoptic phenomenon known as the cold surge is observed (see Section 2.4 for details). Being a coastal urban environment, Bangkok experiences land–sea breezes very frequently.

### 2.2. Data

**Figure 1:**
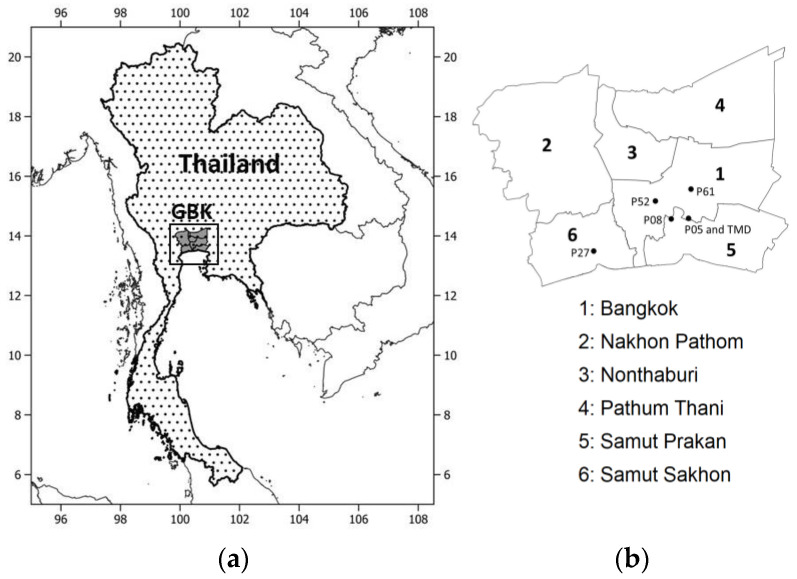
(**a**) Thailand and Greater Bangkok (GBK); (**b**) GBK provinces with PCD and TMD stations. In (**b**) P05, P08, P27, P52 and P61 are the air quality stations at Thai Meteorological Department in Bang Na, Vocational Rehabilitation Center for Disable Person Phra Pradaeng, Samut Sakhon Witthayalai School, Electricity Substation Thonburi, and Bodindecha School, respectively. Thai Meteorological Department (TMD) is the standard weather station (WMO484530) at Bang Na District.

Hourly PM_2.5_ and PM_10_ and other meteorological data, which include air temperature (T), wind speed (WS), wind direction (WD), relative humidity (RH), and global radiation (GR) from five air quality stations (P05, P08, P27, P52 and P61) for the years 2016–2022 were requested and obtained from PCD, which is the main government agency that administers air quality monitoring stations across Thailand (Table 1 and Figure 1b). The selected PCD stations are only located in three provinces in GBK, i.e., Bangkok (No. 1), Samut Prakan (No. 5), and Samut Sakhon (No. 6). During the time of our study, Nakhon Pathom (No. 2) and Nonthaburi (No. 3) did not have PCD stations with PM_2.5_ monitoring. Pathum Thani (No. 4) did, but only for very recent years (Figure 1b). Meteorological data were used based on the availability over different PCD stations as shown in Table 1. Cloud cover (CC) data were obtained for the meteorological station over Bang Na operated by the Thai Meteorological Department (TMD), which is responsible for weather monitoring and forecasting in Thailand. It is noted that the winter season months considered in this study are December, January, and February for the season years 2017–2022. Here, a season year is defined as the period spanning from November of its previous calendar year to October of the year in question. For example, the season year of 2020 covers November 2019 to October 2020. Data were checked for detectable limits or probable ranges for PM_2.5_ and PM_10_ (0 to 1000 µg m^−3^), NO_x_ and SO_2_ (0 to 1000 ppb), CO (0 to 100 ppm), temperature (–5 to 50 °C), relative humidity (0 to 100%), wind speed (0 to 50 ms^−1^), wind direction (0° to 360°), rain (0 to 1000 mm h^−1^) and global radiation (0 to 1000 W m^−2^) [45]. Based on these ranges and manual inspection, we screened for any suspicious or erratic values. Data were checked for missing values and were found to be well within the acceptable range for all variables, except wind speed and wind direction over P05, which were then gap-filled with wind data from the P08 station using linear regression and interpolation methods. The recirculation of air is generally observed over a coastal area due to sea breeze, posing a possible difficult condition for pollutants to be ventilated out of the area and then accumulated. Allwine and Whiteman (1994) [46] proposed a metric called recirculation factor (RF) as the difference between one and the ratio of total wind displacement and total wind run over a period of time as given below:

(1) $$\mathrm{RF}\;=1-\frac{\sqrt{X^{2}+Y^{2}}}{\mathrm{WR}}$$

(2) $$\mathrm{WR}\;=T\;\sum_{i=1}^{N}\sqrt{X^{2}+Y^{2}}$$

(3) $$X\;=T\;\sum_{i=1}^{N}u_{i}$$

(4) $$Y\;=T\;\sum_{i=1}^{N}v_{i}$$

where WR is the wind run computed as the summed distance over which an air mass travels, and X and Y are the total distances traveled by the air mass in the east–west and north–south directions, respectively. N is the total hours of air–mass migration (here, 1000–1800 LT as the daytime hours), and T is the time interval (here, one hour).

Aerosol optical depth (AOD) data at 500 nm were obtained from the Advanced Himawari Imager (AHI) on board Himawari-8. Himawari-8 is the geostationary meteorological satellite by the Japan Meteorological Agency (JMA) [47]. Here, we used the Level 3 version 3.1 hourly AOD_merged_ product available at a spatial resolution of 0.05°. The AOD data were downloaded from the P-Tree System of the Japan Aerospace Exploration Agency (JAXA) Himawari Monitor website (https://www.eorc.jaxa.jp/ptree/index.html, accessed on 10 September 2022). Daily AOD was computed as the average AOD between 09 and 17 LT. Sea surface temperature (SST) data for the Gulf of Thailand were retrieved from European Centre for Medium Weather Forecasting Reanalysis-5 (ERA5; spatial resolution: 0.25° × 0.25°) reanalysis product available from the Climate Data Store (https://cds.climate.copernicus.eu/, accessed on 10 September 2022). Both satellite and reanalysis datasets were downloaded for December, January and February of the season years 2017–2022.

### 2.3. Haze Days and Episodes

**Figure 2:**
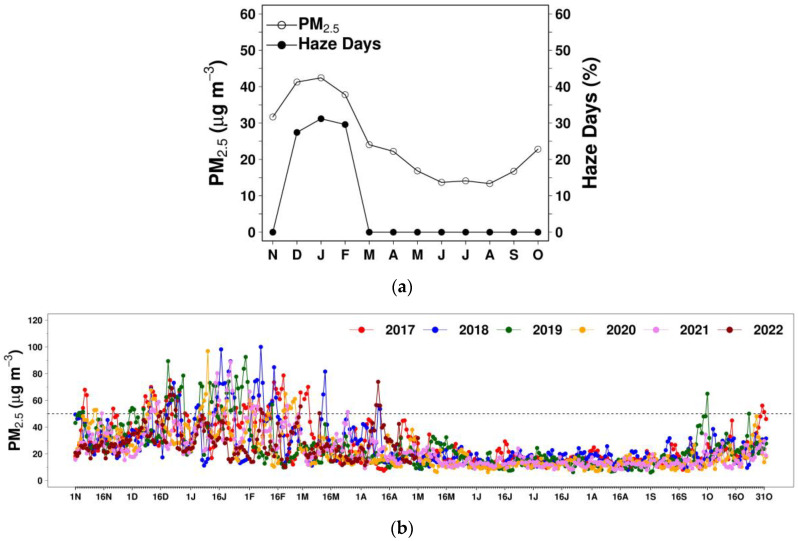
(**a**) Monthly variation for PM_2.5_ and haze days over selected PCD stations over the season years 2017–2022; (**b**) daily PM_2.5_ for Greater Bangkok. The x-axis labels (N, D, J, F, M, ..., S and O) denote the months of the year from November to October.

Haze refers to an atmospheric condition when the air pollutant concentration in the atmosphere is elevated to a higher level than on a normal day. Here, a haze day is defined as a day with 24 h average PM_2.5_ (shortly, daily PM_2.5_) exceeding 50 µg m^−3^ for two or more PCD stations. In Figure 2a,b, both daily PM_2.5_ and haze days are higher during the dry season as compared to the wet season, which typically intensifies during the winter season in December–February and justifies our focus on these three months. Haze episodes were defined as the period of consecutive haze days. Based on visual inspection, non-haze days (1–2 days generally) were also included in an episode if these days fell in between two different consecutive periods of haze days.

### 2.4. Cold Surge Identification

A cold surge (CS) is a periodic synoptic phenomenon over East and Southeast Asia which is originated from the development of a high-pressure system over the Siberian–Mongolian region and its southward propagation to mid-latitudes (i.e., mainland China and East Asia) and low-latitudes (i.e., Indochina Peninsula and the equatorial South China Seas). A cold surge does not have any universal definition and is identified based on observing the change or increase/decrease in one or two meteorological variables over a given geographical area of interest. These variables can include change in meridional wind at a certain height [38,48,49], drop in surface temperature [50], change in both meridional wind and temperature [40,51], change in surface pressure, temperature, and wind speed [52]. A simpler way is the qualitative estimation as used by Aman et al. (2020) [7] to identify CS days over GBK by the visual examination of CFSv2-based synoptic charts. We followed Aman et al. (2020) [7] for the identification of CS days using synoptic charts for 13 LT, those are routinely generated by TMD and can be obtained online (https://www.thaiwater.net/weather, accessed on 12 September 2022). The representative synoptic charts for the day with and without cold surge are given in Figure S1 in Supplementary Materials.

### 2.5. Sea Breeze Identification

The identification of the sea breeze (SB) day needs to investigate three criteria: (a) the identification of synoptic condition not suited for sea breeze formation, (b) thermal forcing criterion, and (c) offshore to onshore wind reversing criterion. Borne et al. (1998) [53] developed the filter based on all these criteria, while other studies, e.g., Furberg et al. (2002) [31] did not account for synoptic conditions. Phan and Manomaiphiboon (2012) [33] compared the result with and without considering the synoptic conditions and found that the results agreed with each other over the coastal region of Eastern Thailand. In this study, we followed the criteria as in Furberg et al. (2002) [31]. The criteria for the SB days are as follows: (a) wind blows offshore or calm for the majority of the hours between six hours before sunrise to two hours after sunrise, (b) wind must blow onshore for at least two consecutive hours between two hours after sunrise to two hours after sunset, (c) wind blows offshore for the majority of the hours between two hours after sunrise to eight hours after sunset, (d) the difference in daily daytime (between sunrise and sunset) temperature over land and daily temperature over sea surface being greater than 3 °C. The range for the wind directions for offshore and onshore wind is dependent on the direction of the coastline and was determined subjectively. For SST, the spatially average temperature over 12.9° N–13.4° N and 100.1° E–100.9° E was computed, while for wind and temperature over land, P05 station data were used. Local sunrise and sunset times were obtained from https://www.timeanddate.com/. SB duration and inland penetration were also estimated.

### 2.6. Biomass Burning and Back-Trajectories

To identify biomass burning in Upper Southeast Asia, 1 km resolution active fire product from MODIS (moderate resolution imaging spectroradiometer) sensors onboard both Terra and Aqua satellites (MCD14ML Collection 6) [54] (available at https://firms.modaps.eosdis.nasa.gov/download/, accessed on 15 September 2022) were downloaded. The fire hotspot data were then summed and gridded to 0.25° and daily gridded maps of fire counts were developed. Next, daily kinematic back-trajectories were simulated using the Hybrid Single-Particle Lagrangian Integrated Trajectory model (HYSPLIT) of the National Oceanic and Atmospheric Administration (NOAA) [55], which was run online (https://www.ready.noaa.gov/HYSPLIT.php, accessed on 23 October 2022) using 3-hourly 0.5° resolution global data assimilation system (GDAS) data (2017–2019) and 3-hourly 0.25° global forecast system (GFS) data (2020–2022) for driving meteorological fields. The arrival time and height were set as 1300 LT and 500 m (above ground level), respectively, which represent the time and mid-height of a well-developed atmospheric boundary layer. Next, the potential effect of biomass burning on urban haze in GBK was identified subjectively by visual examination of the daily fire hotspot map and horizontal and vertical migrations of the back-trajectory for each day.

### 2.7. Identification of Different Haze Types

Based on the influence of cold surge and sea breeze and their interaction, haze episodes were grouped into four types as follows:

(a) Type I: Haze episodes are associated with the arrival of cold surges in GBK during the pre-haze period, which brings cool and relatively strong winds. After a few days of its arrival, it creates stagnant air conditions with limited vertical motion and calm winds. These conditions suppress the vertical dilution of air pollutants and are also not transported horizontally, leading to the accumulation of air pollutants and hence the haze conditions. In short, Type I corresponds to “cold-surge influenced”.
(b) Type II: Haze episodes tend to be under the influence of sea breezes from the Gulf of Thailand for most of the days. This category represents haze episodes that are induced by the combined effect of (1) the recirculation of air pollutants by sea breezes, which brings them back to the city after being initially transported away from the city, (2) potentially developed thermal internal boundary layer due to the advection of cool and stable air over the hot land surface. Air is thermally unstable near the Earth’s surface below cool and stable air, which acts as a cap and prevents the vertical mixing of air pollutants. In short, Type II corresponds to “sea-breeze influenced”.
(c) Type III: Haze episodes are synergistically influenced by the cold surge and sea breeze. A typical haze episode is triggered by the arrival of cold surges in the pre-haze period as suggested for Type I, but as cold surges dissipate, the sea breeze starts, which could potentially affect air pollution dispersion as mentioned in Type II. In some episodes, reverse patterns were also observed when SB days were identified for the first few days followed by CS days.
(d) Type IV: Haze episodes are not affected by either cold surges or sea breeze. Most of the haze episodes are short 1-day episodes and are also affected by biomass burning.

### 2.8. Effect of Secondary Aerosols

To examine the potential effects of secondary aerosols on different haze episodes, a precursor-ratio method was adapted from Zhang et al. (2015) [56] and Aman et al. (2019) [8]. Air pollutant data from only one PCD station over Bang Na (P05) were considered due to constraints on data availability over other PCD stations (Table 1). In this method, the ratio of PM_2.5_, SO_2_, and NO*_x_* to CO was computed and then averaged for different haze episodes and then compared with the averaged ratios of these normalized pollutants for the clean days of the year to which an episode belongs. CO is generally emitted by incomplete combustion of carbon-containing fuels and has a relatively long chemical lifetime as compared to other typical air pollutants. Hence, it can be used as a tracer for the primary emission. An increase in the PM_2.5_/CO and a decrease in SO_2_/CO, NO*_x_*/CO potentially suggest more chemical conversion from the gas phase to the particle phase (sulfur dioxide to sulfate and nitrogen oxides to nitrate) and an increase in the formation of secondary aerosols. Daily average concentrations for different pollutants were decided based on the diurnal variation of these pollutants.

## 3. Results

### 3.1. Temporal Characteristics of Identified Haze Days and Haze Episodes

**Figure 3:**
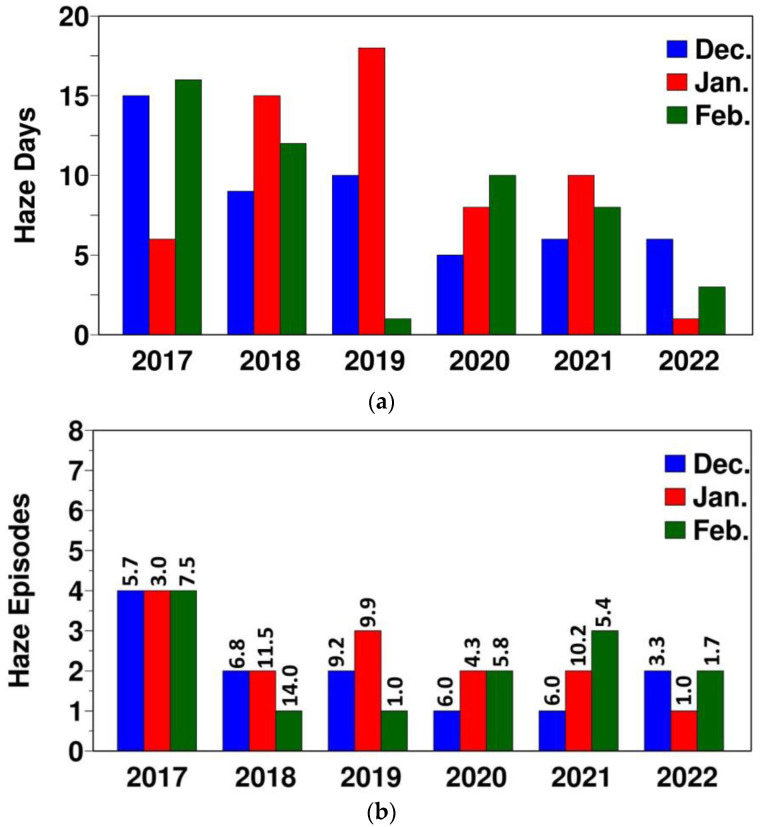
(**a**) Month-to-month variation. (**a**) Haze days; (**b**) haze episodes. In (**b**), the value above each bar is the average haze duration.

The month-to-month variations in the total number of haze days and haze episodes over 2017–2022 are given in Figure 3a,b, respectively. In the 6 years, a total of 159 haze days were found over Greater Bangkok. Based on the visual examination of these haze days, they were grouped into 38 haze episodes (Table S1 in Supplementary Materials). These haze episodes either have all consecutive haze days or have 1–2 clean days in between but had pollution buildup again and were under a similar kind of haze type. These 38 haze episodes comprise a total of 169 days. Overall, the total number of haze days and haze episodes decreased during 2017–2022 (Figure 3).

The frequency of haze episodes against haze duration is shown in Table 2. The minimum duration of a haze episode is 1, while the maximum duration is 14. Short episodes of 1–2 days are the most common, which occurred 18 times. The frequency of haze episodes decreases with an increase in haze durations. A total of eight persistent haze (defined here as haze duration >7 days) episodes were found, and six of them were classified as Type III, which represents haze under the synergetic effect of the cold surge and sea breeze (see details of haze types in Section 3.2). The short- and medium-range haze episodes (defined here as haze duration ≤ 7 days) were mostly under other haze types, which were influenced by either cold surges or sea breeze or neither of them. Additionally, the coefficient of variation, i.e., the ratio of standard deviation to the mean of daily PM_2.5_ during persistent haze episodes is relatively higher as compared to other haze episodes, suggesting more day-to-day variation in PM_2.5_ likely due to the complex formation mechanism. Various aspects of these four haze types are described in the next section.

### 3.2. Characteristics of Different Haze Types

**Figure 4:**
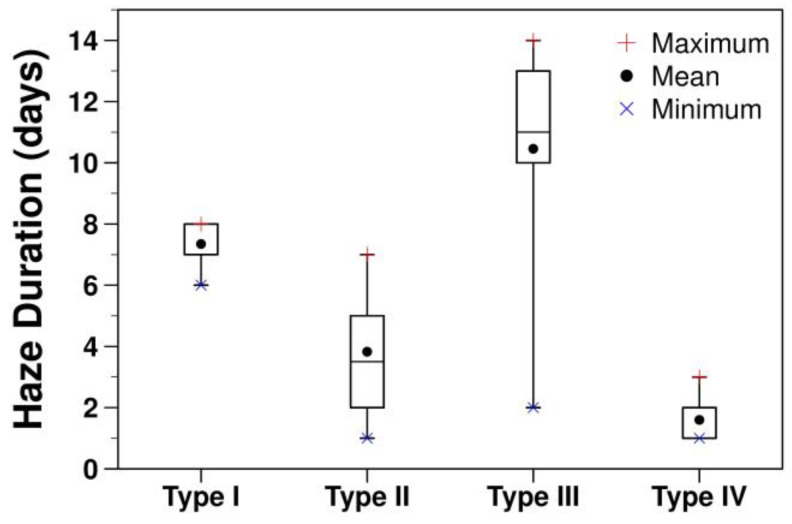
Haze duration by haze type.

**Figure 5:**
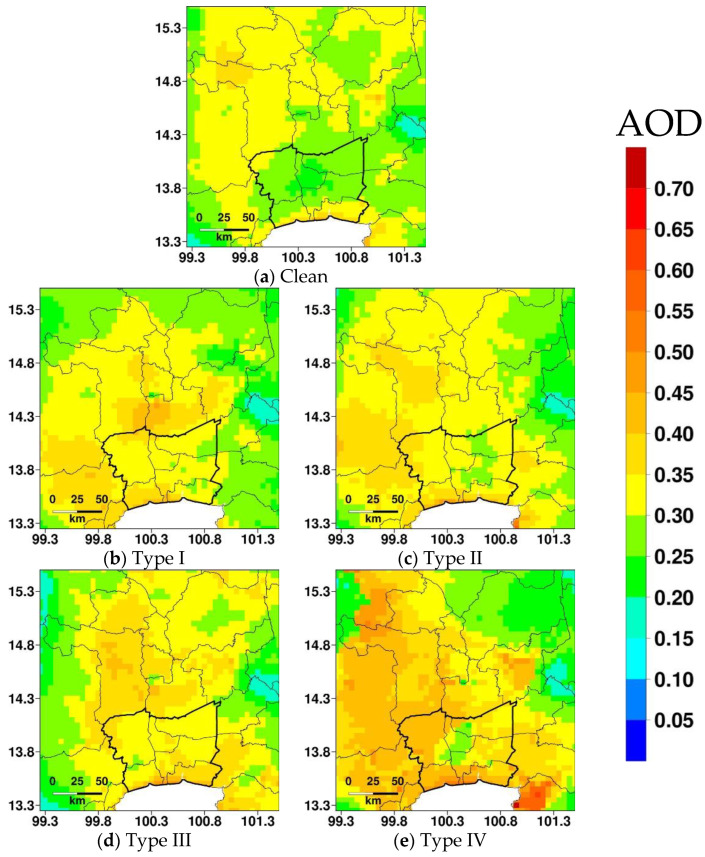
Average AOD at 500 nm from AHI over Greater Bangkok (bordered by the thick lines) and its vicinity by haze type.

The overview of haze durations, pollution levels, and meteorological conditions in four haze types are given in Table 3 and Figure 4 and Figure 5 and are summarized below:

(a) Type I: Four haze episodes are categorized in this haze type. This is the second most persistent haze type, and the average duration of haze episodes is 7.3 days. It has two haze episodes of eight days and a haze episode each of six and seven days, respectively (Table 2). In terms of particulate pollution, this is the second most polluted haze type with an average PM_2.5_ of 58.5 μg m^−3^. As this haze type is triggered by the cold surge over Greater Bangkok, which brings cold and dry air, it has the lowest average temperature and average relative humidity (26.9 °C and 49.1%, respectively). A cold surge generally induces less cloud cover, which leads to an increase in global radiation as shown by the lowest average cloud cover of 4.9 and the second-highest average global radiation of 456.6 W m^−2^. Due to more stagnant weather conditions, wind recirculation is relatively low with an average recirculation factor of 0.12. The spatial variation of AOD suggests relatively higher AOD (ranges between 0.2 and 0.6) in Greater Bangkok and connected provinces on the northern and western sides as compared to those on the eastern side and far north. In comparison, AOD over clean days is relatively low over Greater Bangkok.
(b) Type II: This is the most frequent haze type with 15 haze episodes. The average minimum and maximum durations of haze episodes are 3.8 days, 1 day, and 7 days respectively. Nine of the fifteen haze episodes are of only 1–2 days. The average PM_2.5_ is 56.8 μg m^−3^. As this haze type is influenced by SB, which brings humid air from the seaside, the highest average RH of 59.1% is reported, while the average temperature is 29.1 °C. The second-lowest average cloud cover and highest average global radiations of 5.1 and 471.1 W m^−2^, respectively, were reported. Due to the influences of sea breeze, a relatively higher average recirculation factor of 0.3 is found. The average SB duration and SB penetration are 7.6 h and 31.6 km, respectively. Compared with Type I, the aerosol loading is higher at a larger distance from Greater Bangkok and over larger regions, which can be attributed to the advection of air pollutants.
(c) Type III: This is the second most frequent haze type with 11 haze episodes and the most persistent type with an average haze episode duration of 10.5 due to the synergetic effect of the cold surge and sea breeze. This is the most polluted haze type with an average PM_2.5_ of 62.8 μg m^−3^. The average values for temperature, relative humidity, cloud cover, global radiation, and recirculation factor are 27.6 °C, 58.1%, 5.2, 453.5 W m^−2^, and 0.3, respectively. Due to the interaction of the sea breeze with the cold surge from the opposite direction, the average SB duration and SB penetration are lower than those in type II (6.2 h and 20.5 km, respectively). The spread of haze or aerosols is over a larger area in the north, as suggested by higher AOD, which can be attributed to both the advection and dispersion of air pollutants, given multiple persistent haze episodes.
(d) Type IV: This category consists of eight haze episodes, and seven of them are one-day episodes, while the remaining one is a three-day episode. The average PM_2.5_ under this haze type is 54.1 μg m^−3^. Many of the haze episodes are associated with biomass burning. The effect of biomass burning on different haze episodes is discussed and illustrated in Section 3.4. The average values for temperature, relative humidity, cloud cover, global radiation, and recirculation factor are 28.4 °C, 58.2%, 5.5, 414.1 W m^−2^, and 0.2, respectively. The sea breeze was reported on the last day of the 3-day haze episode with duration and penetration of 12 h and 36.2 km, respectively. The spread of aerosol is over larger regions as suggested by higher AOD over the region, which can be attributed to biomass burning.

### 3.3. Effect of Secondary Aerosols

Based on the diurnal variation of PM_2.5_, NO*_x_*, SO_2_, and CO (results not shown), only daytime hours (here, 06–18 LT) were considered. The selected hours include early morning hours when the pollutants concentration represents the fresh emission due to increased anthropogenic activities and later hours when the chemical and photochemical reaction occurs. The average values for PM_2.5_/CO, NO*_x_*/CO, and SO_2_/CO for the 13 episodes that are potentially affected by secondary aerosol formation are given in Table 4. The average value of PM_2.5_/CO is higher during episodic days as compared to the clean days for all the episodes. Both NO*_x_*/CO and SO_2_/CO decrease during episodic days in five episodes, which suggests the potential formation of both sulphate and nitrate PM. Eight episodes showed a drop in only NO*_x_*/CO, suggesting the formation of only nitrate particles. Six out of these thirteen episodes are induced by sea breezes (Type II haze), which are also associated with relatively higher relative humidity, which could play important role in the formation of secondary aerosols. However, the effect of secondary aerosols is also found in the other three haze types. These results suggest the potential role of secondary aerosol in haze formation, but it may not be present in all cases.

### 3.4. Effect of Biomass Burning

The details of the episodes that are potentially affected by biomass burning are given in Table 5. A total of 86 days out of 541 days between 2017 and 2022 were identified to have wind trajectories pass over fire hotspot areas before reaching Bangkok, hence potentially contributing to air pollution in GBK. However, this potential contribution seems to be significant only for 42 days when haze develops in Bangkok. A total of 19 episodes out of 38 seem to have limited to-serve effects on haze episodes based on the assumption that a minimum of one day during a haze episode should have the wind trajectory coming from fire hotspots regions. Based on the trajectories and fire hotspots examination, the effect of biomass burning was classified into two types: (a) low impact, and (b) high impact. The haze episodes for which the number of days with biomass burning effect was less than 50% of the total duration of haze episodes were considered to have a low impact or otherwise. The high impact is found in over 10 episodes, which are short haze episodes of 1–2 days. For the remaining nine medium-to-long-duration haze episodes, the effect of biomass burning is in complement with the local anthropogenic emission and stagnant weather conditions. It should be noted that many days which were classified to have the effect of biomass burning do not have a haze over Bangkok due to favorable conditions for the dilution of the air pollutants.

**Figure 6:**
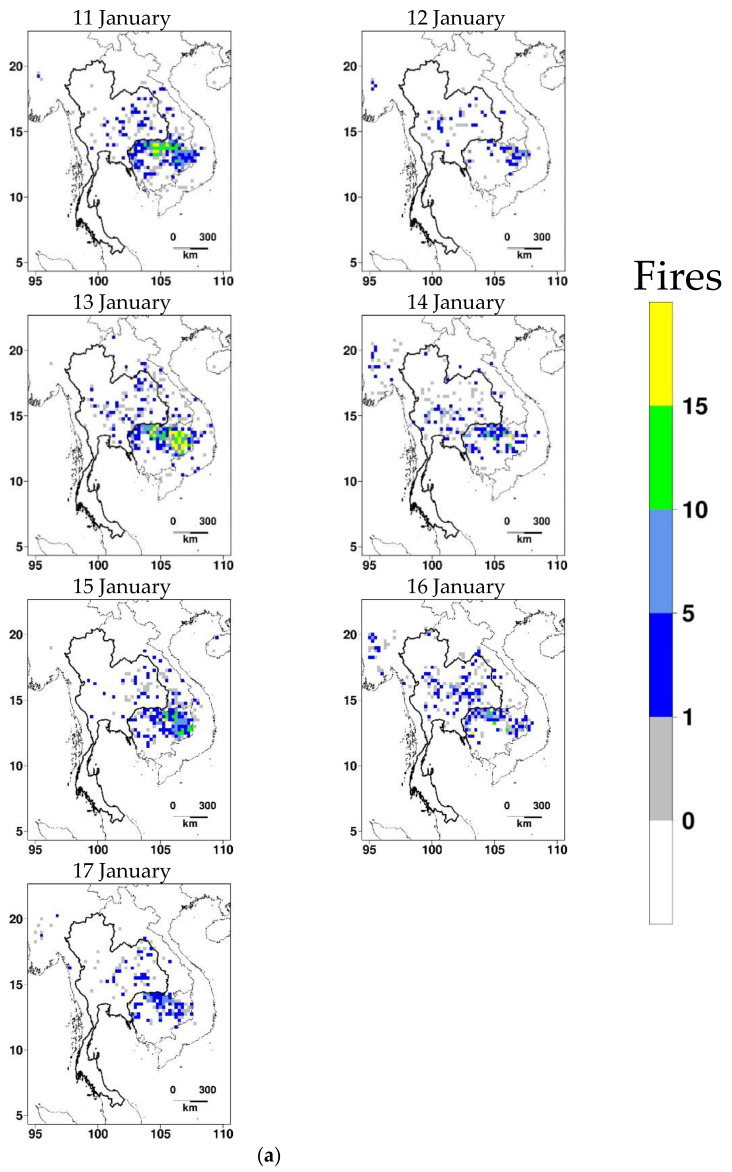

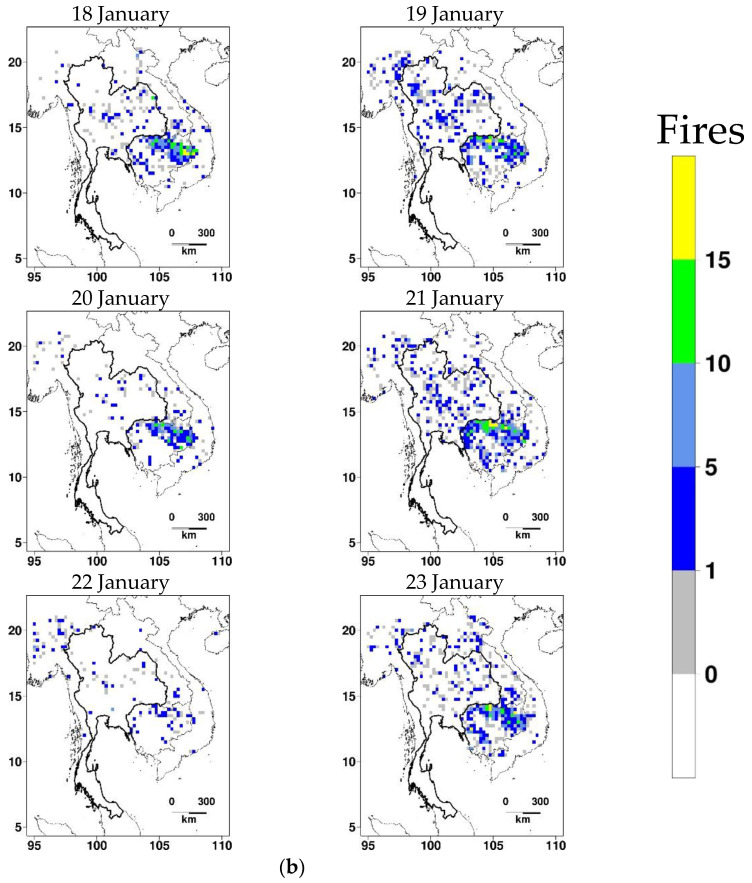
Daily fire counts on 0.25° cells during (**a**) Episode 20 and a few days afterward; (**b**) Episode 25 and a few days afterward.

**Figure 7:**
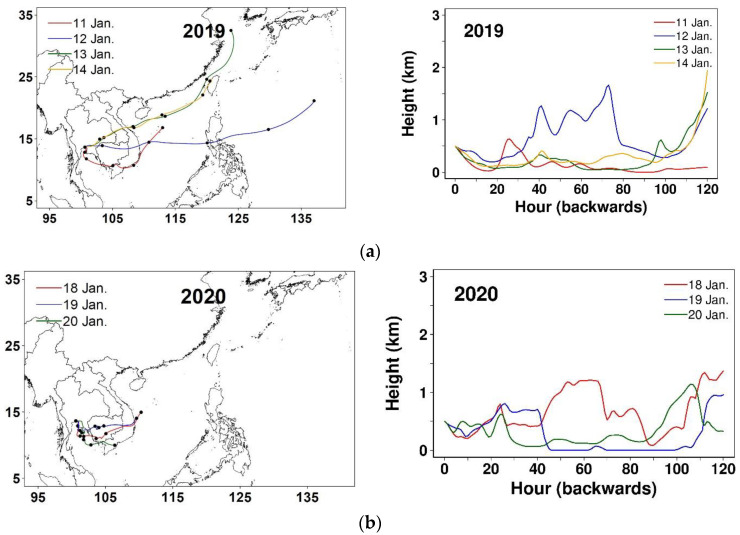
Horizontal (**left**) and vertical migration (**right**) of daily 120 h back-trajectories during (**a**) Episode 20; (**b**) Episode 25.

For illustration, the fire hotspot maps and daily 120 h back-trajectories for Episode 20 (low impact) and Episode 25 (high impact) are displayed in Figure 6 and Figure 7, respectively. Episode 20 spans four days (11–14 January 2019), classified as Type II (i.e., under cold surge alone). Fires are more seen in Cambodia than in Thailand throughout the episode (and also a few days after). No back-trajectories pass over the dense fire areas before reaching GBK, except on the second day. However, the corresponding back-trajectory does not stay at low levels. Episode 25 spans three days (18–20 January 2020), classified as Type IV (not under either cold surge or sea breeze). Like the previous episode, fires occur more intensely in Cambodia. Although fires in Cambodia may potentially influence haze in the study during the first two days, only the second day has the back-trajectory moving slowly at low levels over the dense fire areas.

## 4. Discussion

Motivated by the importance of winter-time haze and its association with meteorology, this study presents an effort to review recent haze episodes in GBK using the meteorological classification for haze with cold surge and sea breeze as the central framework. The findings from the investigation and analysis indicate that winter-time haze involves multiple factors, such as emissions, meteorology (at local, meso-, and synoptic scales), and secondary aerosols. Short haze episodes tend to couple with the cold surge or sea breeze alone, but extended haze episodes can experience and couple with the cold surge and sea breeze individually or combined. Prolonged haze can permit favorable conditions for secondary aerosol formation. GBK has a very large number of emission sources. Transport (particularly on-road) and industries are the typical major sectors. Nevertheless, the influence of biomass burning (within, outside, or distant from the study area) and secondary aerosols are sometimes non-negligible. Based on the precursor-ratio method applied here, 13 episodes (out of 38) are potentially affected by secondary aerosols. Narita et al. (2019) [17] also reported secondary aerosols as a contributor to haze in the study area using source apportionment. As found here, a number of the haze episodes considered were impacted by biomass burning to varying degrees. Aman et al. (2020) [7] investigated the day-to-day evolution of two winter-time haze episodes with contrasting impacts of biomass burning. Phairuang et al. (2019) [18] reported a significant contribution from agricultural burning in Central Thailand and forest fires in the north to particulate pollution in Bangkok during winter. Dejchanchaiwong et al. (2020) [19] found fires in Thailand and Cambodia to be a contributor to a haze episode in Bangkok. Aman et al. 2022 [20] analyzed two low-visibility events in Bangkok, both of which were partly affected by biomass burning.

Some policy implications related to haze in GBK can be derived from the findings in this study: (1) Many haze episodes are well associated with the synoptic (cold surge) and mesoscale (sea breeze) processes, posing a challenge to haze mitigation by emission reduction. (2) Secondary aerosols contribute to haze, needing to control their gaseous precursors. (3) Biomass burning, though not considered a major contributor in all haze episodes, should also be managed, indicating a need for a comprehensive emission database to support the design and implementation of measures to cope with haze properly.

## 5. Conclusions

In this study, haze characteristics under the influence of cold surge and sea breeze were investigated using the observed data over Greater Bangkok (GBK) in 2017–2022, including haze intensity and duration, and meteorology-based haze classification, as well as the potential effects of secondary aerosols and biomass burning. A total of 159 haze days and 38 haze episodes were identified. Haze duration (per episode) varies from a single day to many days (up to 8–14 days), the latter of which signifies the long-lasting persistence of haze and also indicates different pathways of formation and evolution of haze episodes. Eighteen haze episodes are short-duration episodes of 1–2 days, and the frequency of haze episodes decreases with an increase in haze duration. The larger values of the coefficient of variation of PM_2.5_ in relatively long episodes suggest the increased complexity in the haze formation and evolution. The four haze types (Types I–IV) were identified, accounting for cold surges, sea breeze, and their coupling. Both cold surges and sea breeze are two important atmospheric processes that influence winter-time weather conditions for the study area, particularly December–February. In Type I, a haze episode is triggered by the arrival of the cold surge in the pre-haze period, with stagnant weather that is favorable for haze later. Type II corresponds to haze induced by the sea breeze with pollutants accumulated likely due to local recirculation and the thermal internal boundary layer. For Type III, haze is driven by the synergetic effect of the cold surge and sea breeze. For Type IV, haze is neither under cold surge influence nor under sea breeze influence. Type II is the most frequently occurring haze type with a total of 15 episodes. Type III has the highest average duration of 10.8 days with six persistent haze episodes, while Type IV has mostly single-day haze episodes. Type III is also the most polluted haze type due to the synergetic effect of the cold surge and sea breeze and haze spreading broadly beyond GBK in the north and northeast directions due to advection and dispersion. Type I is the driest and coolest due to the effect of cold surge, while Type II is the most humid with the highest recirculation factor due to the highest average sea breeze duration and penetration. Type IV has mostly single-day episodes, and higher AOD is found over larger regions near GBK possibly due to biomass burning. The precursor ratio method applied indicates the potential effect of secondary aerosols for 13 haze episodes (34%). Using the visual examination of back-trajectories and fire hotspots, biomass burning can be readily seen to affect 19 episodes (50%), but the high impact is mostly constrained to the relatively short haze episodes, while the relatively long haze episodes correspond to a mixed impact from biomass burning, local emissions, and meteorology.

Future work to further the understanding of haze in the study area can be extended to the following topics: quantified contributions of meteorology and emissions to haze [57], health impact of air pollution [58], vertical evolution of haze and atmospheric stability during cold surges and sea breezes, [59], quantitative meteorological classification [60], episodic chemical characterization and source apportionment, and the spatiotemporal variation of haze with satellite-based PM_2.5_ mapping using statistical and machine learning modeling [61]. It is also useful to incorporate more PM_2.5_ data into the analysis if the monitoring network becomes more extensive over the study area. Given that the sea breeze was shown to influence haze, its associated parameters should be incorporated into predictive models for haze and tested.

## Figures and Tables

**Table 1:** Surface stations and variables used in the study.

Station	Coordinates (Latitude, Longitude)	Province	Variables	Background
P05	13.67, 100.61	Bangkok	PM_2.5_, PM_10_, NO*x*, SO_2_, CO, T, RH, WS, WD	General area
P08	13.66, 100.54	Samut Prakan	WS, WD	General area
P27	13.55, 100.26	Samut Sakhon	PM_2.5_, PM_10_, T, GR	Mixed (roadside and general area)
P52	13.73, 100.49	Bangkok	PM_2.5_, PM_10_, T, RH	Roadside
P61	13.77, 100.61	Bangkok	PM_2.5_, PM_10_, T	General area
TMD (WMO484530)	13.67, 100.61	Bangkok	CC	General area

Remark: PM_2.5_ and PM_10_ (μg m^−3^): Particulate matter with size not larger than 2.5 µm and 10 µm, respectively; NO*_x_* (ppb): nitrogen dioxide; SO_2_ (ppb): sulphur dioxide; CO (ppm): carbon monoxide; T (°C): temperature; WS (m s^−1^): wind speed at 10 m; WD (deg. from the north): wind direction at 10 m; RH (%): relative humidity; GR (W m^−2^): global radiation; CC (fraction, ranging from 0 to 10 as the cloud-free and fully clouded conditions, respectively): cloud cover. The stations have terrain elevations of 2–4 m above mean sea level.

**Table 2:** Number of haze episodes by haze durations.

Duration (Days)	No. of Haze Episodes	No. of Episodes by Haze Type (I-II-III-IV)	Coefficient of Variation of Daily PM_2.5_ (Standard Deviation/Mean)
1	11	0-4-0-7	0.1
2	7	0-5-2-0	0.16
3	3	0-2-0-1	0.1
4	3	0-1-2-0	0.12
5	2	0-2-0-0	0.21
6	2	1-0-1-0	0.1
7	2	1-1-0-0	0.09
8	2	2-0-0-0	0.15
10	2	0-0-2-0	0.16
11	1	0-0-1-0	0.29
13	2	0-0-2-0	0.21
14	1	0-0-1-0	0.25
Total	38	4-15-11-8	

**Table 3:** PM_2.5_ and different meteorological variables (mean ± standard deviation) by haze type.

Variable	Clean	Type I	Type II	Type III	Type IV
PM_2.5_ (μg m^−3^)	31.3 ± 10.1	58.4 ± 1.8	56.8 ± 5.7	62.8 ± 6.4	54.1 ± 5.8
T (°C)	27.9 ± 1.5	26.9 ± 2.2	29.1 ± 0.7	27.6 ± 1.1	28.4 ± 1.3
RH (%)	64.1 ± 11.8	49.1 ± 4.8	59.1 ± 5.7	58.1 ± 6.3	58.2 ± 9.7
CC (Fraction)	6.0 ± 1.7	4.9 ± 1.5	5.1 ± 1.2	5.2 ± 1.5	5.5 ± 2.2
GR (W m^−2^)	428.6 ± 89.6	456.6 ± 36.9	471.1 ± 24.5	453.5 ± 40.5	414.1 ± 102.5
RF (unitless)	0.1 ± 0.2	0.1 ± 0.1	0.3 ± 0.2	0.3 ± 0.1	0.2 ± 0.1
SB Duration (h)	7.6 ± 4.0	NA	7.6 ± 3.2	6.2 ± 2.6	12.0
SB Penetration (km)	36.5 ± 35.8	NA	31.6 ± 25.3	20.5 ± 17.1	36.2

**Table 4:** Average PM_2.5_/CO, NO*_x_*/CO, and SO_2_/CO during the haze episodes potentially affected by secondary aerosols.

Episode	Type	PM_2.5_/CO		NO_x_/CO		SO_2_/CO	
		Clean	Episodic	Clean	Episodic	Clean	Episodic
1	Type I	56.5	57.2	55.9	53.1	2.9	2.5
6	Type II	56.5	63.6	55.9	54.5	2.9	2.7
7	Type IV	56.5	66.3	55.9	50.6	2.9	3.6
11	Type IV	56.5	83.8	55.9	52.2	2.9	2.9
12	Type II	66.4	79.1	59.9	54.5	2.7	4.2
16	Type III	66.4	83.4	59.9	50.61	2.7	2.5
21	Type III	52.8	57.1	50.3	48.6	1.7	1.8
22	Type II	52.8	63.7	50.3	44.8	1.7	1.7
25	Type IV	56.9	67.2	57.8	46.9	1.8	0.9
26	Type II	56.9	61.6	57.8	48.6	1.8	1.5
27	Type I	56.9	66.3	57.8	38.1	1.8	2.3
37	Type II	47.0	51.7	36.7	31.2	2.1	–
38	Type II	47.0	56.2	36.7	35.9	2.1	–

**Table 5:** Effect of biomass burning on different haze episodes.

Episode	Duration (Days)	No. of Days Affected by Biomass Burning (Day Number)	Impact
04	1	1 (1st)	High
07	1	1 (1st)	High
08	5	1 (2nd)	Low
10	10	4 (1st to 4th)	Low
11	1	1 (1st)	High
15	13	4 (4th, 6th, 8th and 13th)	Low
16	14	5 (1st, 3rd, 4th, 10th and 14th)	Low
18	10	1 (6th)	Low
19	2	2 (1st and 2nd)	High
20	4	1 (2nd)	Low
21	13	4 (4th, 5th, 7th and 9th)	Low
24	5	(2nd and 5th)	Low
25	3	2 (1st and 2nd)	High
26	3	2 (2nd and 3rd)	High
27	7	6 (1st, 2nd, 3rd, 4th, 5th and 6th)	High
30	11	2 (4th and 9th)	Low
32	1	1 (1st)	High
33	2	1 (1st)	High
37	2	2 (1st and 2nd)	High

## Data Availability

The data presented in this study are available on request from the corresponding author.

## Supplementary Materials

The following supporting information can be downloaded at: https://www.mdpi.com/article/10.3390/ijerph20043482/s1, Table S1: List of the identified haze episodes and their corresponding types. Figure S1: Example of representative synoptic charts for (a) Days with cold surge; (b) Days without cold surge, with focus on GBK and the central region of Thailand.
