# Supplementary material for: A Study of Urban Haze and Its Association with Cold Surge and Sea Breeze for Greater Bangkok
Source: Int J Environ Res Public Health. 2023 Feb 16;20(4):3482. doi: 10.3390/ijerph20043482 (PMC9964824; doi:10.3390/ijerph20043482)
Supplement: Supplementary file 1 [file ijerph-20-03482-s001.zip › ijerph-2158384-supplementary.pdf]

A Study of Urban Haze and Its Association with Cold Surge and Sea Breeze for Greater Bangkok (Supplementary Materials)

Table S1. List of the identified haze episodes and their corresponding types.

Episode	Period	Type	Duration (days)
01	07/12/2016 – 14/12/2016	I	8
02	18/12/2016 – 21/12/2016	III	4
03	25/12/2016 – 26/12/2016	II	2
04	29/12/2016	IV	1
05	07/01/2017	IV	1
06	15/01/2017	II	1
07	25/01/2017	IV	1
08	29/01/2017 – 02/02/2017	II	5
09	05/02/2017 – 07/02/2017	II	3
10	10/02/2017 – 19/02/2017	III	10
11	28/02/2017	IV	1
12	10/12/2017 – 11/12/2017	II	2
13	19/12/2017 – 26/12/2017	I	8
14	04/01/2018 – 05/01/2018	II	2
15	12/01/2018 – 24/01/2018	III	13
16	02/02/2018 – 15/02/2018	III	14
17	01/12/2018	IV	1
18	19/12/2018 – 28/12/2018	III	10
19	06/01/2019 – 07/01/2019	III	2
20	11/01/2019 – 14/01/2019	II	4
21	19/01/2019 – 31/01/2019	III	13
22	13/02/2019	II	1
23	10/12/2019 – 15/12/2019	I	6
24	06/01/2020 – 10/01/2020	II	5
25	18/01/2020 – 20/01/2020	IV	3
26	01/02/2020 – 03/02/2020	II	3
27	19/02/2020 – 25/02/2020	I	7
28	10/12/2020 – 15/12/2020	III	6
29	06/01/2021	II	1
30	13/01/2021 – 23/01/2021	III	11
31	01/02/2021 – 07/02/2021	II	7
32	21/02/2021	IV	1
33	26/02/2021 – 27/02/2021	II	2
34	15/12/2021 – 16/12/2021	III	2
35	21/12/2021 – 24/12/2021	III	4
36	07/01/2022	IV	1
37	07/02/2022 – 08/02/2022	II	2
38	28/02/2022	II	1

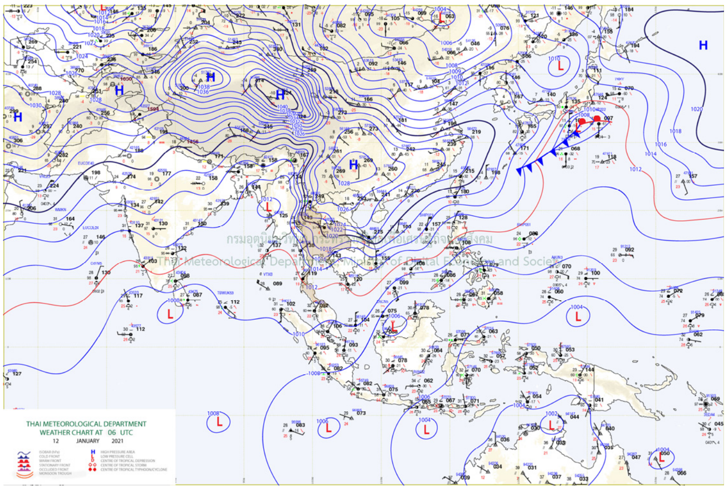

(a)

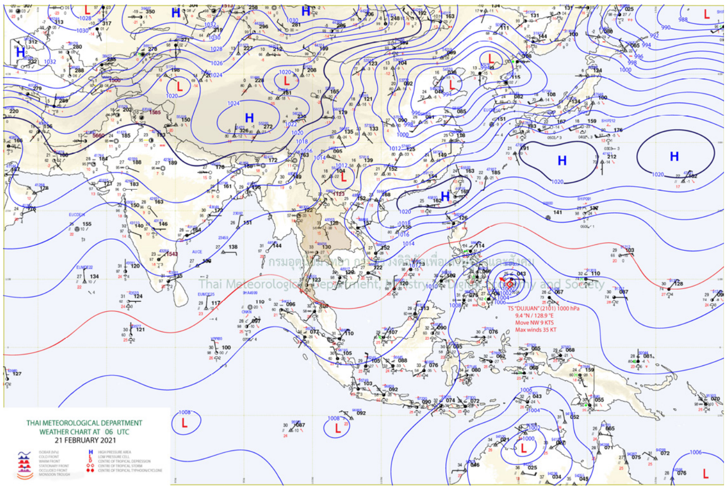

(b)

Figure S1: Example of representative synoptic charts for **(a)** Days with cold surge; **(b)** Days without cold surge, with focus on GBK and the central region of Thailand.
